# Validity of an Abbreviated, Clinically Feasible Test for Postprandial Lipemia in Healthy Adults: A Randomized Cross-Over Study

**DOI:** 10.3390/nu11010180

**Published:** 2019-01-16

**Authors:** Christina M. Sciarrillo, Nicholas A. Koemel, Stephanie P. Kurti, Sam R. Emerson

**Affiliations:** 1 Department of Nutritional Sciences, Oklahoma State University, Stillwater, OK 74078, USA; christina.sciarrillo@okstate.edu (C.M.S.); nick.koemel@okstate.edu (N.A.K.); 2 Department of Kinesiology, James Madison University, Harrisonburg, Virginia, VA 22807, USA; kurtisp@jmu.edu

**Keywords:** fat tolerance, triglycerides, post-meal, risk assessment

## Abstract

Background: A large post-meal triglyceride (TG) response is an independent risk factor for cardiovascular disease, but postprandial lipemia assessments are not clinically practical in their current form. Therefore, we assessed the validity of an abbreviated, clinically feasible protocol in measuring postprandial lipemia. Method: Eighteen healthy adults (8 male and 10 female) completed 3 high-fat meal trials in random order: (1) a Standard in Lab (SL) protocol wherein blood draws (to determine TG) were made from a catheter at baseline and hourly for 6 h; (2) an Abbreviated in Lab (AL) protocol in which participants remained in the laboratory but blood draws were only made at baseline and 4 h post-meal; and (3) an Abbreviated with Freedom (AF) protocol in which participants vacated the laboratory between the meal and the 4-h blood draw. Results: TG increase from baseline was very similar (*p* = 0.93) across the 3 trials (SL: 68.5 ± 62.7 mg/dL; AL: 71.1 ± 58.0 mg/dL; AF: 66.7 ± 46.4 mg/dL), as were 4-h TG levels (SL: 144.6 ± 84.2 mg/dL; AL: 171.4 ± 88.2 mg/dL; AF: 157.7 ± 76.7 mg/dL; *p* = 0.49). Similarly, total and incremental area under the curve (AUC) were not significantly different across the trials (*p* = 0.12 and 0.91, respectively). Conclusion: The TG results of the clinically feasible, abbreviated protocol were similar to those of the more exhaustive standard protocol. The AF protocol could be a valid and feasible clinical tool for measurement of postprandial lipemia and assessment of cardiovascular risk, although studies in larger and more diverse cohorts are needed.

## 1. Introduction

A large post-meal triglyceride (TG) response (“postprandial lipemia”) is a risk factor for cardiovascular disease (CVD) and a stronger predictor of risk than fasting TG values [1]. Additionally, many individuals in Western society spend the majority of their day in a non-fasted or postprandial state [2]. However, postprandial lipemia assessments are not clinically practical in their current form, requiring numerous blood draws and participants to stay in the laboratory for several hours. Research studies will typically measure TG via hourly blood draws for 6–8 h post-meal e.g., [3]. Only a few studies have aimed to simplify the process of assessing postprandial lipemia [3,4,5]. Findings from a previous study suggest that an abbreviated protocol, in which TG are only measured for 4 h post-meal, may be a valid surrogate for assessing the entire postprandial response [6]. The validity of a single postprandial TG measurement at 4-h post-meal has been subsequently supported by findings from Rector et al. [4] and Maraki et al. [5]. Nevertheless, these studies utilize a procedure that still requires participants to remain sedentary in the laboratory between the baseline and postprandial TG measurements. In our view, it is not reasonable to necessitate patients to stay in a clinic for 4 consecutive hours, therefore more efforts are needed to develop a clinically feasible test for postprandial lipemia.

The purpose of this study was to assess the validity of an abbreviated, clinically feasible protocol in measuring the post-meal TG response in young, healthy adults. We hypothesized that allowing participants to leave the laboratory between the baseline and 4-h follow-up blood draw would not significantly alter TG results. This hypothesis was based in part on null findings from studies that investigated whether postprandial exercise of moderate energy expenditure modifies the TG response curve (e.g., [7]).

## 2. Methods

### 2.1. Research Participants

We recruited 8 men and 10 women to participate in this study. Inclusion criteria were age 18–45 years and free of chronic disease, confirmed via medical history questionnaire. Individuals were excluded if they were pregnant, taking lipid-lowering medications, or using tobacco. All participants gave their informed consent for inclusion before they participated in the study. The study was conducted in accordance with the Declaration of Helsinki, and the protocol (HE1757) was approved by the Institutional Review Board at Oklahoma State University.

### 2.2. Overview of Study

**Figure 1:**
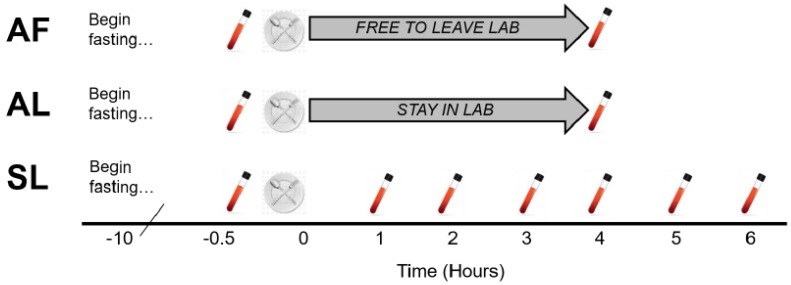
Protocol for the three meal trials. Participants completed 3 meal trials in random order. The Standard in Lab (SL) trial featured blood draws from a venous catheter at baseline and hourly for 6 h post-meal. The Abbreviated in Lab (AL) trial included single blood draws at baseline and 4 h post-meal, while the participants remained in the lab. In the Abbreviated with Freedom (AF) trial, participants vacated the lab between the meal and the 4-h blood draw.

Participants completed 3 meal test protocols in random order: Standard in Lab (SL), Abbreviated in Lab (AL), and Abbreviated with Freedom (AF). The SL trial was intended to be representative of standard postprandial testing procedures commonly used in experimental protocols, therefore, blood was drawn at baseline and every hour for 6 h via venous catheter while participants remained sedentary in the laboratory. The AL trial was intended to be representative of the abbreviated protocol tested by Weiss et al. [6], therefore, only 2 blood draws were obtained, at baseline and 4 h post-meal and participants remained in the laboratory during the assessment. In the AF trial, only 2 blood draws were made, but participants were allowed to leave the laboratory between meal consumption and the 4-h blood draw. Each meal trial is depicted in Figure 1.

### 2.3. Study Protocol

For 3 days prior to their first assessment, participants recorded their dietary intake using a food log, and then replicated their food consumption prior to each of the remaining trials. Participants also avoided planned exercise for 2 days before each meal test. Meal trials were separated by at least 1 week.

Participants consumed a 210-kcal snack consisting of whole-grain peanut butter crackers (Snyder’s-Lance, Inc., Charlotte, NC, USA) ten hours prior to each meal trial (i.e., the evening before). The purpose of the snack was to ensure consistency in the fast across trials and participants. In the SL trial, due to the frequency of blood draws, an intravenous (IV) catheter was set in a forearm vein and kept patent by slow infusion of 0.9% NaCl. A baseline/fasting blood draw was then made from the IV catheter. In the AL and AF trials, blood draws were conducted via single venipuncture. In all trials, blood samples were ultimately collected in a 6 mL test tube coated with EDTA (Ethylene Diamine Tetra-acetic Acid). Whole blood was then inserted into a Cholestech LDX Lipid + Glu cassette and processed using a Cholestech LDX analysis system to determine metabolic outcomes (Alere Cholestech; Hayward, CA, USA). The coefficient of variation for TG using Cholestech is 2–4%.

Following the baseline blood draw, participants then consumed the high-fat meal (HFM), which consisted of Marie Callender’s Chocolate Satin Pie (Conagra Brands; Omaha, NE, USA), as we have used previously [8]. The HFM was normalized to body weight (12 kcal/kg; 63% fat, 34% carbohydrate). Participants consumed the test meal in <20 min and water was provided *ad libitum* during the meal and postprandial period.

In the SL and AL trials, participants remained sedentary in the laboratory. In the AF trial, participants were instructed to: (1) leave the laboratory; (2) not consume anything besides water; and (3) avoid planned/structured exercise, although activities of daily living were permissible.

### 2.4. Statistical Analyses

Weiss et al. [6] tested the validity of an abbreviated lipemia protocol (essentially the AL trial in the present study) in a cohort of 9 individuals (2 M/7 F). In this cohort, the authors found solid evidence in support of the abbreviated 4-h test. To be safe, we aimed to recruit 15 individuals, with a maximum enrollment of 20 participants. Data were checked for normality via the Shapiro-Wilk normality test. Area under the curve (AUC) was calculated using the trapezoidal method. Fasting TG, 4-h TG, 4-h TG Δ (from baseline/fasting), total AUC (tAUC represents the overall postprandial TG response), and incremental AUC (iAUC represents the postprandial TG response above fasting levels) were compared across the 3 trials using a one-way analysis of variance (ANOVA) or Friedman test (for non-parametric data), as well as calculation of 95% confidence intervals. To understand whether the 4-h time-point reasonably captured the TG peak, 3-h versus 4-h TG levels in the SL trial were compared via paired *t*-test, and time-to-peak for the SL trial was determined via AUC analysis. To determine whether validity of the abbreviated protocols was influenced by sex or BMI, post hoc secondary analyses (one-way ANOVA) were conducted in which the sample was divided by sex (males and females) and BMI (normal weight and overweight/obese). Statistical analyses and figure generation were conducted via GraphPad Prism 7 (Graphpad Software, Inc., La Jolla, CA, USA).

## 3. Results

Eighteen individuals, 8 male (M) and 10 female (F), participated in the present study (Table 1). Eleven participants (3 M/8 F) were healthy weight (18.5–24.9 kg/m^2^), three participants (3 M) were overweight (25–29.9 kg/m^2^), and four participants (2 M/2 F) were obese (>30 kg/m^2^). Five participants reported with fasting TG > 150 mg/dL on at least one occasion, although no participants reported with fasting TG > 180 mg/dL.

**Figure 2:**
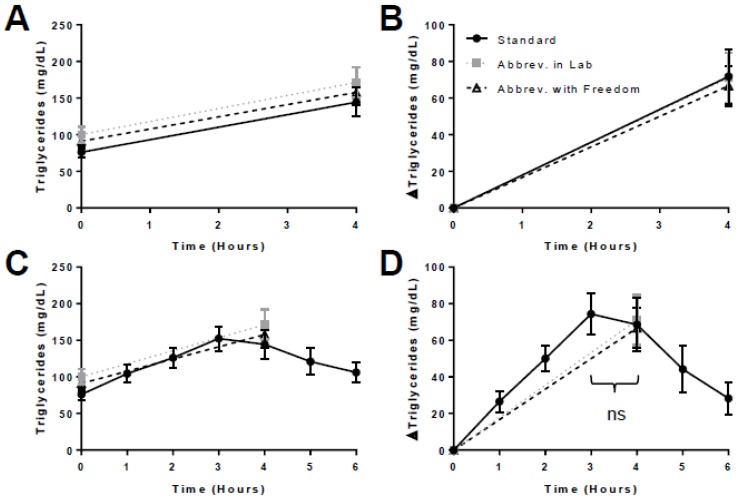
Postprandial triglyceride responses in the 3 meal trials. For clarity, postprandial triglyceride data are presented in four ways (**A**–**D**), varying whether data are normalized for fasting values (Panels **B**,**D**) and overlaid with hourly data from the SL trial (Panels **C**,**D**). There were no significant differences in the postprandial triglyceride response across the 3 trials. Additionally, “ns” denotes that there was no difference between the 3-h and 4-h triglyceride levels in the SL trial.

There was no difference in fasting TG, 4-h TG, 4-h TG Δ, tAUC, or iAUC across the 3 trials (Table 2). The postprandial TG responses were very similar across all 3 trials (Figure 2), especially when normalized for fasting values (Figure 2B). When overlaid with hourly data from the SL trial (Figure 2C,D), the response curves suggest that the 4-h time-point generally captured the TG peak. There was no difference in 3-h versus 4-h TG in the SL trial (*p* = 0.65). Across participants, the TG peak occurred at 3.7 ± 1.2 h in the SL trial. When men and women were analyzed separately, there were still no differences across trials in fasting TG (men, *p* = 0.44; women, *p* = 0.11), 4-h TG (men, *p* = 0.84; women, *p* = 0.08), or 4-h TG Δ (men, *p* = 0.39; women, *p* = 0.33). Similarly, when normal weight and overweight/obese individuals were analyzed separately, there were no differences across trials in fasting TG (normal weight, *p* = 0.28; overweight/obese, *p* = 0.23), 4-h TG (normal weight, *p* = 0.39; overweight/obese, *p* = 0.50), or 4-h TG Δ (normal weight, *p* = 0.61; overweight/obese, *p* = 0.81).

## 4. Discussion

The purpose of this pilot study was to assess the validity of a novel abbreviated and clinically feasible test for postprandial lipemia in a group of young, healthy adults. We hypothesized that allowing participants to leave the laboratory between the baseline and 4-h follow-up blood draw would not significantly alter TG results. Our results supported this hypothesis, as we did not observe statistical differences between the 3 trials with regard to the postprandial indices utilized. Additionally, our results suggest that measurement of the 4-h time-point reasonably captured the TG peak. Collectively, these findings support the notion of an abbreviated protocol for testing postprandial lipemia, including allowing participants to leave the laboratory during the test.

Bansal et al. found that non-fasting/postprandial triglycerides were a stronger predictor of CVD risk than fasting values [1]. In fact, these authors found that adjustment for confounding variables eliminated the association between fasting TG and CVD incidence, while non-fasting TG remained an independent CVD predictor even after adjustment [1]. However, relatively little effort has been made to translate postprandial TG assessments to a clinical setting. In 2001, Guerci and colleagues [3] found that a simplified postprandial protocol, in which only 3 blood draws were made, correlated with the conventionally measured TG response. In 2008, Weiss et al. [6] observed that an abbreviated, 4-h postprandial lipemia test was a valid surrogate for the entire postprandial response—although this was assessed statistically, not by actually conducting an abbreviated test. These findings were subsequently supported by findings from Rector et al. [4] and Maraki et al. [5], and in 2011, an expert panel statement supported the notion of a single TG measurement 4 h post-HFM, suggesting it may be useful for clinical testing [9]. Very recently, O’Doherty and colleagues [10] found strong evidence for the repeatability of a 4-h TG test.

Our finding of similar TG responses between the SL and AF trials represents an innovative and novel next step toward clinical applicability of postprandial lipemia testing. The AF protocol, which requires only two blood draws and allows participants to leave the laboratory, is much more clinically feasible than the standard postprandial lipemia protocol, as it greatly reduces both the extensive time and blood-work requirements associated with standard postprandial assessments. We believe that allowing patients to leave the clinic for ~4 h greatly enhances the feasibility of assessing postprandial lipemia. Given that the SL and AF trials yielded highly similar results, a possible downstream implication of this work is that more individuals could receive a postprandial TG measurement as a strategy for assessing CVD risk, potentially allowing for more timely lifestyle or pharmacological intervention. In addition, utilization of such a protocol in research could open up study designs that would have otherwise been viewed as too cumbersome.

However, certain limitations need to be considered when interpreting our data. The present investigation was a pilot study with a relatively small sample size. Additionally, we did not conduct an a priori sample size estimation, but rather determined our target sample size based on previous, similar investigations [6,10]. Lastly, we did not control for menstrual cycle phase, which may have influenced our findings for female participants.

Much work remains with regard to determining the clinical utility of the AF protocol. The validity of the AF protocol needs to be tested in larger cohorts of individuals, especially in groups across the CVD risk spectrum. In addition, the reproducibility of the AF protocol needs to be elucidated. Finally, testing the AF protocol in a clinical setting will be critical and informative in ultimately determining its potential clinical use.

## Figures and Tables

**Table 1:** Participant characteristics.

**Participants (*n*)**	18
Age (years)	21.2 ± 2.5
Sex (M/F)	8 M/10 F
Body mass (kg)	72.9 ± 16.9
Height (cm)	169.7 ± 2.9
BMI (kg/m^2^)	25.2 ± 6.1
Fasting glucose (mg/dL)	90.8 ± 7.6
Fasting Total-C (mg/dL)	165.9 ± 33.5
Fasting HDL-C (mg/dL)	53.3 ± 19.3
Fasting LDL-C (mg/dL)	95.1 ± 27.9
Test meal energy (kcal)	874.8 ± 202.7

Fasting metabolic markers represent an average of fasting levels across the three trials. Data are Mean ± SD. M, male; F, female; BMI, body mass index; Total-C, total cholesterol; HDL-C, high-density lipoprotein cholesterol; LDL-C, low-density lipoprotein cholesterol.

**Table 2:** Fasting triglyceride levels and postprandial indices.

		SL Trial	AL Trial	AF Trial	*p*
Fasting TG (mg/dL)	Mean ± SD	76.1 ± 34.0	100.4 ± 44.6	91.1 ± 44.9	0.08
	95% CI	(59.2, 93.0)	(78.2, 122.6)	(68.7, 113.4)	
4-h TG (mg/dL)	Mean ± SD	144.6 ± 84.2	171.4 ± 88.2	157.7 ± 76.7	0.49
	95% CI	(102.7, 186.4)	(127.6, 215.3)	(119.6, 195.8)	
4-h TG Δ (mg/dL)	Mean ± SD	68.5 ± 62.7	71.1 ± 58.0	66.7 ± 46.4	0.93
	95% CI	(37.3, 99.7)	(42.2, 99.9)	(43.6, 89.7)	
tAUC (mg/dL × 4 h)	Mean ± SD	441.2 ± 224.2	543.7 ± 254.5	497.6 ± 233.5	0.12
	95% CI	(329.7, 552.7)	(417.1, 670.2)	(381.4, 613.7)	
iAUC (mg/dL × 4 h)	Mean ± SD	138.6 ± 123.6	143.2 ± 114.5	133.3 ± 92.7	0.91
	95% CI	(77.1, 200.0)	(86.3, 200.2)	(87.2, 179.4)	

Data are represented as Mean ± SD and 95% confidence intervals. The *p* value column represents the findings of a one-way analysis of variance (ANOVA). There were no significant differences when assessed by ANOVA or 95% confidence intervals. SL, standard in lab; AL, abbreviated in lab; AF, abbreviated with freedom; TG, triglycerides; tAUC, total area under the curve; iAUC, incremental area under the curve.
